# Ultrasonic Incisions Produce Less Inflammatory Mediator Response during Early Healing than Electrosurgical Incisions

**DOI:** 10.1371/journal.pone.0073032

**Published:** 2013-09-18

**Authors:** Bindu Nanduri, Ken Pendarvis, Leslie A. Shack, Ranjit Kumar, Jeffrey W. Clymer, Donna L. Korvick, Shane C. Burgess

**Affiliations:** 1 Department of Basic Sciences, College of Veterinary Medicine, Mississippi State University, Mississippi State, Mississippi, United States of America; 2 Department of Veterinary Science and Microbiology, College of Agriculture and Life Sciences, Tucson, Arizona, United States of America; 3 Center for Clinical and Translational Science, University of Alabama at Birmingham, Birmingham, Alabama, United States of America; 4 Preclinical Research, Ethicon Endo-Surgery, Inc., Cincinnati, Ohio, United States of America; 5 College of Agriculture and Life Sciences, University of Arizona, Tucson, Arizona, United States of America; Virginia Commonwealth University School of Medicine, United States of America

## Abstract

As the use of laparoscopic surgery has become more widespread in recent years, the need has increased for minimally-invasive surgical devices that effectively cut and coagulate tissue with reduced tissue trauma. Although electrosurgery (ES) has been used for many generations, newly-developed ultrasonic devices (HARMONIC® Blade, HB) have been shown at a macroscopic level to offer better coagulation with less thermally-induced tissue damage. We sought to understand the differences between ES and HB at a microscopic level by comparing mRNA transcript and protein responses at the 3-day timepoint to incisions made by the devices in subcutaneous fat tissue in a porcine model. Samples were also assessed via histological examination. ES-incised tissue had more than twice as many differentially-expressed genes as HB (2,548 vs 1,264 respectively), and more differentially-expressed proteins (508 vs 432) compared to control (untreated) tissue. Evaluation of molecular functions using Gene Ontology showed that gene expression changes for the energized devices reflected the start of wound healing, including immune response and inflammation, while protein expression showed a slightly earlier stage, with some remnants of hemostasis. For both transcripts and proteins, ES exhibited a greater response than HB, especially in inflammatory mediators. These findings were in qualitative agreement with histological results. This study has shown that transcriptomics and proteomics can monitor the wound healing response following surgery and can differentiate between surgical devices. In agreement with clinical observations, electrosurgery was shown to incur a greater inflammatory immune response than an ultrasonic device during initial iatrogenic wound healing.

## Background

In any internal surgery, cutting through tissue is required either to gain access to the target organs or to modify those organs. A key objective of any surgical procedure is to minimize collateral iatrogenic damage, incurring the minimum amount of tissue damage necessary to effect the desired repair. The less trauma inflicted, the better the chances for rapid wound healing, with less pain and risk of complications. For incisions through tissue, either non-energized (“cold”) or energized devices are used. Although cold steel scalpels perform an excellent job of making surgical incisions with a low level of iatrogenic trauma compared to energized devices [1], separate hemostasis is necessary. This is especially problematic as laparoscopic procedures have become more popular. Electrosurgery, or electrocautery, provides an efficient method of hemostasis [2], but suffers several drawbacks such as risk of electrical shock, obstructed viewing of the surgical site by smoke, and lateral tissue damage due to the high temperatures from the passage of electrical current. Recently, incision devices have been developed that utilize ultrasonic energy, such as the HARMONIC SYNERGY® Blade (Ethicon Endo-Surgery, Inc., Cincinnati, OH) [3]. This device simultaneously cuts and coagulates by means of high frequency (55.5 kHz) longitudinal oscillation of the blade. Hemostasis is achieved at lower temperatures than those generated by electrosurgery or lasers, so less lateral tissue damage is incurred.

The differences in tissue injury between electrosurgery and ultrasonic have been studied at a macroscopic level. Ultrasonic incisions in rabbit muscle resulted in less infiltration of markers of acute inflammation (e.g., neutrophils) during wound healing than electrosurgical incisions [4]. Ultrasonic incisions in murine muscle also showed less inflammation and faster decrease in fibrosis than electrosurgery [5]. In a study showing reduced effect of ultrasonic incisions on nerve physiology, reduced leukocyte infiltration and axonal transport impairment was observed compared to electrosurgical incisions [6].

However, the underlying molecular functions can be both descriptive and prognostic of the inflammatory process and healing after tissue damage. Although histopathological techniques, combined with reductionist discipline-specific approaches have proved insensitive to describing differences, contemporary functional genomics technologies (transcriptomics and proteomics), which measure the response of genes underlying physiology and pathophysiology, combined with computational modeling techniques, can identify patterns that explain the observed clinical differences in wound healing. Previous microarray studies have examined the effects of simple thermal injury to porcine skin at 48 hours [7] and 7 days [8], but did not attempt to monitor changes in protein levels, nor to detect differences between energized surgical instruments. This is important because although mRNAs are sometimes directly correlated with protein levels, mRNAs do not directly determine phenotype. Proteins in contrast are the direct determinants of phenotype. It is important to measure both, and doing so allows understanding of how a phenotype is regulated and how it may be perturbed; in this case understanding gene expression at both the mRNA and protein levels could be useful in further device design improvements, methods of use, or ancillary medications that could improve healing.

In addition to the availability of microarray chips for porcine genes, analytical methods have recently been developed to survey the porcine proteome [9]. In this study we sought to determine whether we could observe differences in gene transcription or protein levels between energized devices and cold scalpel incisions in porcine subcutaneous adipose tissue at three days after surgery. Further we hoped to learn whether differences between electrosurgery (ES) and ultrasonic (HB) that are observed on a macroscopic level could be discerned at the gene and protein level. Via examination of individual transcripts and proteins, and modeling of differentially expressed genes, our results clearly demonstrate that incisions with energized devices increase the amounts of inflammatory mediators on a molecular level consistent with macroscopic observation of pathology, and that in general electrosurgery produces a greater inflammatory response than ultrasonic incisions.

## Results

### Clinical and Histopathology

**Figure 1:**
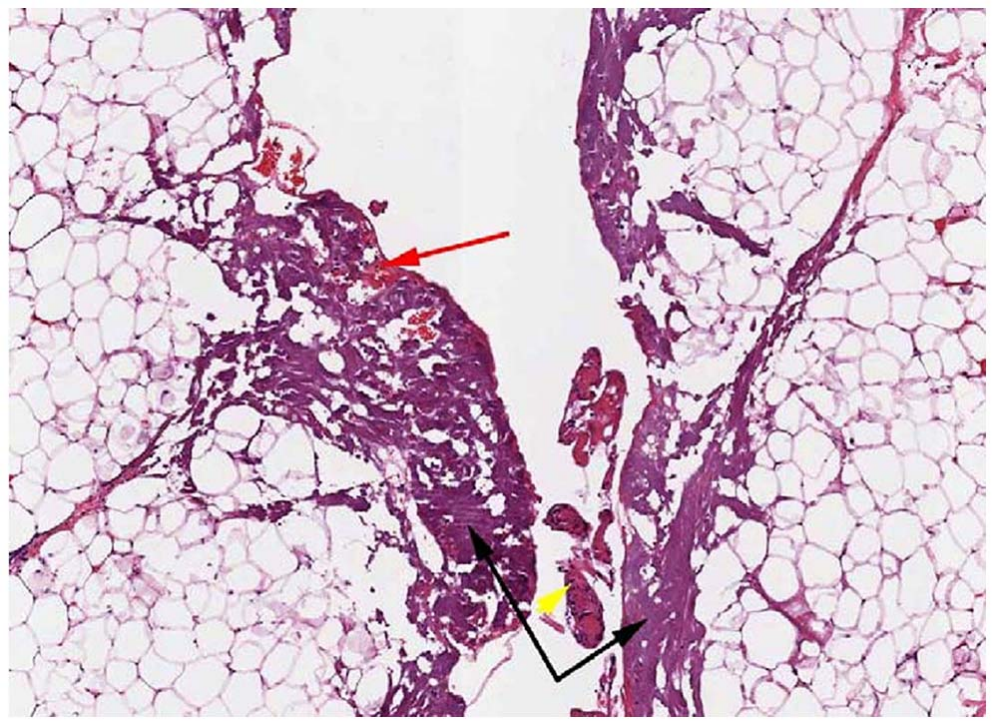
Histopathology analysis revealed mild degeneration/necrosis of the adipose and/or connective tissue (black arrows), minimal acute inflammation (yellow arrow) and minimal hemorrhage (red arrow) are observed in the adjacent tissue surrounding the incision by harmonic blade. The images were acquired at 50X magnification after hematoxylin and eosin staining.

**Figure 2:**
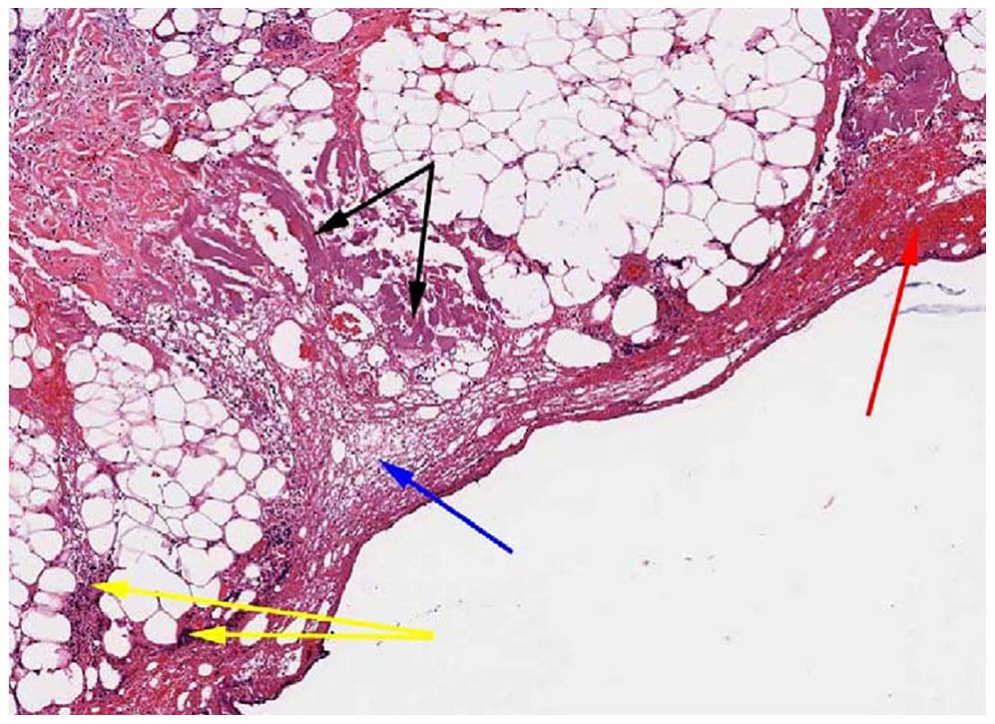
Histopathology analysis revealed mild degeneration/necrosis of the adipose and/or connective tissue (black arrows), mild acute inflammation (yellow arrows), mild fibrin infiltration (blue arrow) and minimal hemorrhage (red arrow) are noted within the connective tissue/fat immediately surrounding the incision by electrosurgery. The images were acquired at 50X magnification after hematoxylin and eosin staining.

Animals recovered normally from surgery with no external signs of inflammation or drainage at the incision sites, except for a small amount of serous fluid. Via histological evaluation, degeneration/necrosis, acute inflammation, fibrin infiltration and hemorrhage were rated as minimal to mild, and granulation tissue formation was rated as absent to minimal for both devices (Figures 1, 2). Average incision depth was 3.2 cm and average incision width was 0.25 cm. Via Mann-Whitney tests, there were no significant differences in the quantitative measures of inflammation. Qualitatively, the ES group displayed a slightly higher severity of inflammatory reaction, fibrin infiltration and granulation tissue formation, and slightly lower severity of degeneration/necrosis than the HB. Incision depth was slightly greater for HB than ES. Within each device there were no apparent differences between samples based on incision depth, although the small number of samples and range of depths precluded a statistical analysis.

### Microarray analysis

**Figure 3:**
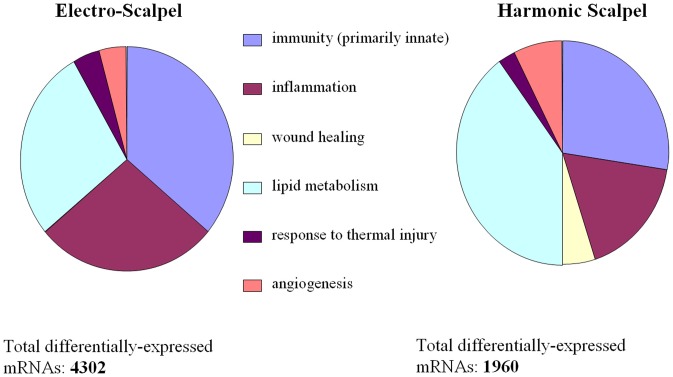
Summary of the biological processes as described by Gene Ontology, represented in the differentially expressed genes in response to harmonic blade and electrosurgery incisions.

**Figure 4:**
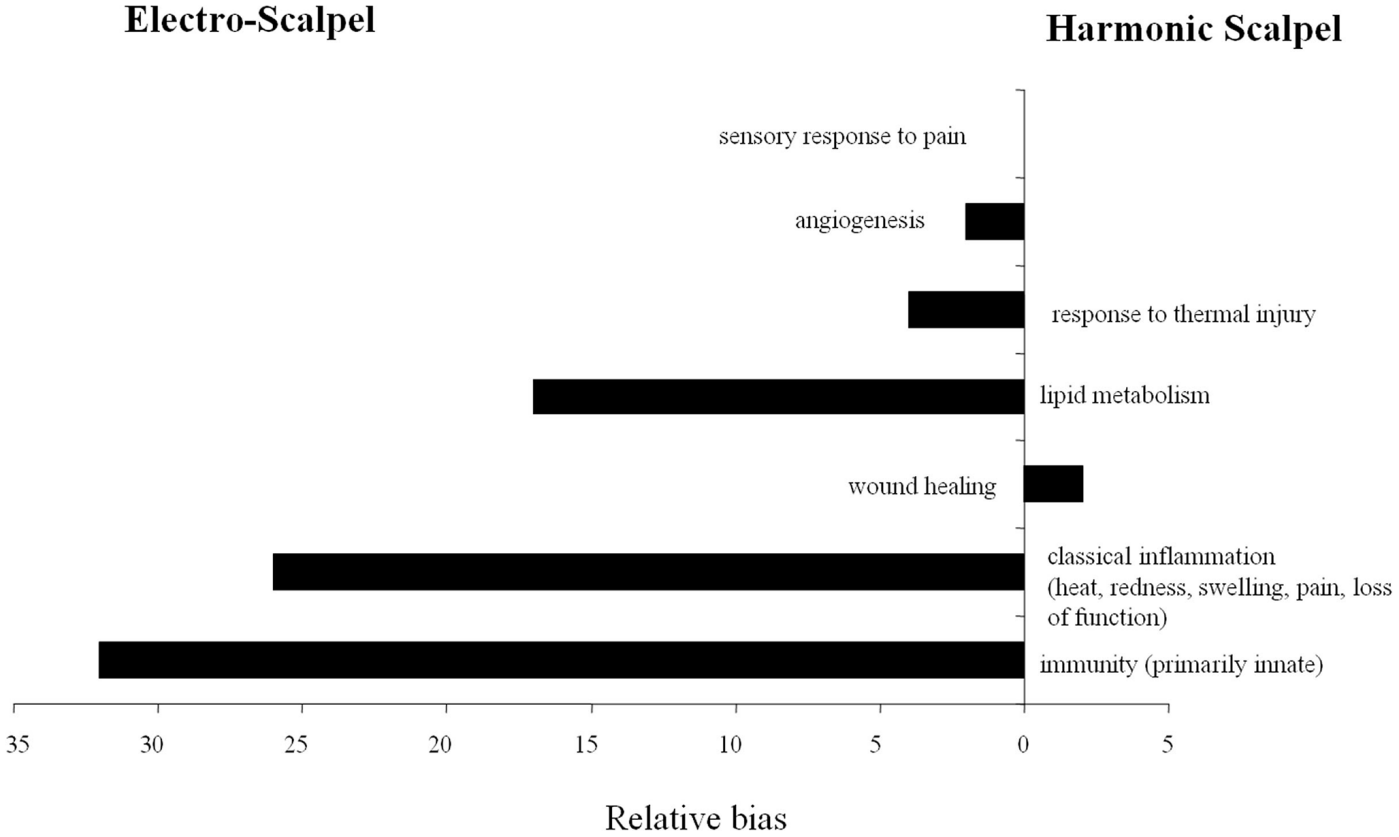
Comparison of the overall effect of the identified gene expression changes in response to harmonic blade and electrosurgery incisions on biological processes of relevance to wound healing using GOModeler workflow. We used Gene Ontology terms to represent specific biological processes, and identify the known effect on a function under consideration such as ‘positively regulates’ or ‘negatively regulates’. The magnitude of the changes in gene expression was also taken into account.

Statistical analysis of the microarray data at the three-day time point indicated that ES-incised tissue had more than twice as many differentially expressed (DE) probes compared to the tissue incised with HB (6,605 vs. 2,414 respectively; table S1A–B). After removing probe, porcine gene Id level redundancies, 2,548 and 1,264 genes were identified as DE (table S1 C–D) in response to ES-incision and HB-incision of porcine tissue some of which are shown in Table 1. For functional modeling of DE genes, all porcine genes were annotated to the Gene Ontology (GO) using the tools available at AgBase [10]. Based on precedence in wound healing literature, we evaluated genes annotated to specific GO categories (Figure 3) for identifying the differences in porcine gene expression in response to two different methods of incisions under study. Although HB-incised tissue had half as many genes differentially expressed compared to ES, genes annotated to wound healing were identified in this dataset. Both types of incisions resulted in DE of genes relevant to tissue trauma such as inflammation, angiogenesis and response to thermal injury. Comparison of the distribution functional categories in each DE dataset showed that ES had a higher proportion of genes involved in inflammation compared to HB. This GO-based summary of DE genes summarizes functions, but does not incorporate the quantitative aspects of DE (up-and down-regulated genes) to evaluate the net effect of these changes on any of these functional categories. We used AgBase tool GOModeler [11] to determine the net effect of DE of genes on these GO terms. GOModeler navigates the GO to determine whether genes annotated to specific function are known to be positive (‘pro’) or negative (‘anti’) regulators of that function, if the information is available, and combines this with quantitative values to determine the net effect on a function. Relative bias of DE in response to ES and HB based on GoModeler analysis showed that there was a positive effect on wound healing in HB (Figure 4). While GO-based analysis represented porcine specific functional information, using human orthologs of porcine genes, we conducted Ingenuity pathways analysis of DE genes to identify significant pathways and networks represented by these genes.

**Table 1:** Up-regulated genes in response to Electrosurgery and Harmonic incisions.

Gene Symbol	Description	ES/C	Corrected p value	HB/C	Corrected p value
CYP1B1	Cytochrome P450 1B1	2.86	6.22E-06	13.9	1.53E-03
TIMP1	Metalloproteinase inhibitor 1	11.1	9.97E-03	7.5	6.43E-04
PPBP	Platelet basic protein precursor (CXCL7)	33.7	4.19E-03	19.1	1.74E-03
IL8	Interleukin-8 (CXCL8)	110.7	2.93E-03	17.9	1.12E-04
CXCL6	Granulocyte chemotactic protein 2	100.9	1.21E-02	72.1	1.45E-05
GPR68	Sphingosylphosphorylcholine receptor	15.8	1.16E-04	10.3	1.35E-03
SERPINB2	Plasminogen activator inhibitor-2	15.6	6.06E-03	6.9	3.46E-04
ARG1	Arginase 1	85.1	1.03E-02	25.2	1.48E-03
TNC	Tenascin	16.3	1.19E-02	8.0	3.02E-03
PRSS35	Protease, serine, 35	14.6	2.04E-03	12.5	1.70E-03
IL1RN	Interleukin-1 receptor antagonist protein	9.9	1.29E-04	11.2	7.13E-04
SELE	E-selectin	10.3	1.01E-02	4.9	4.10E-03
IL6	Interleukin-6	10.5	2.45E-03	5.5	8.54E-04
ALDH9A1	Aldehyde dehydrogenase	10.9	1.05E-02	6.4	4.15E-05
SDS	L-serine dehydratase	13.5	1.21E-02	6.8	7.36E-04
Q6ZUM6	Dynein, cytoplasmic, heavy polypeptide 2	4.02	8.86E-03	1.7	1.24E-03
CYP1B1	Cytochrome P450 1B1	13.7	8.97E-03	11.4	2.27E-03
MARCO	Macrophage receptor	3.07	1.51E-04	1.7	9.72E-04
PF4	Platelet factor 4 precursor (CXCL4)	22.1	9.76E-03	18.3	4.23E-04
CXCL2	Macrophage inflammatory protein-2	32.5	9.27E-03	12.6	1.02E-04

### Proteomics data analysis

**Figure 5:**
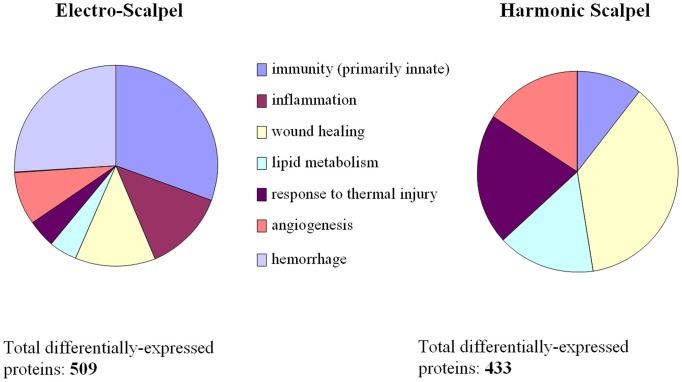
Summary of the biological processes as described by Gene Ontology, represented in the differentially expressed proteins in response to harmonic blade and electrosurgery incisions.

**Figure 6:**
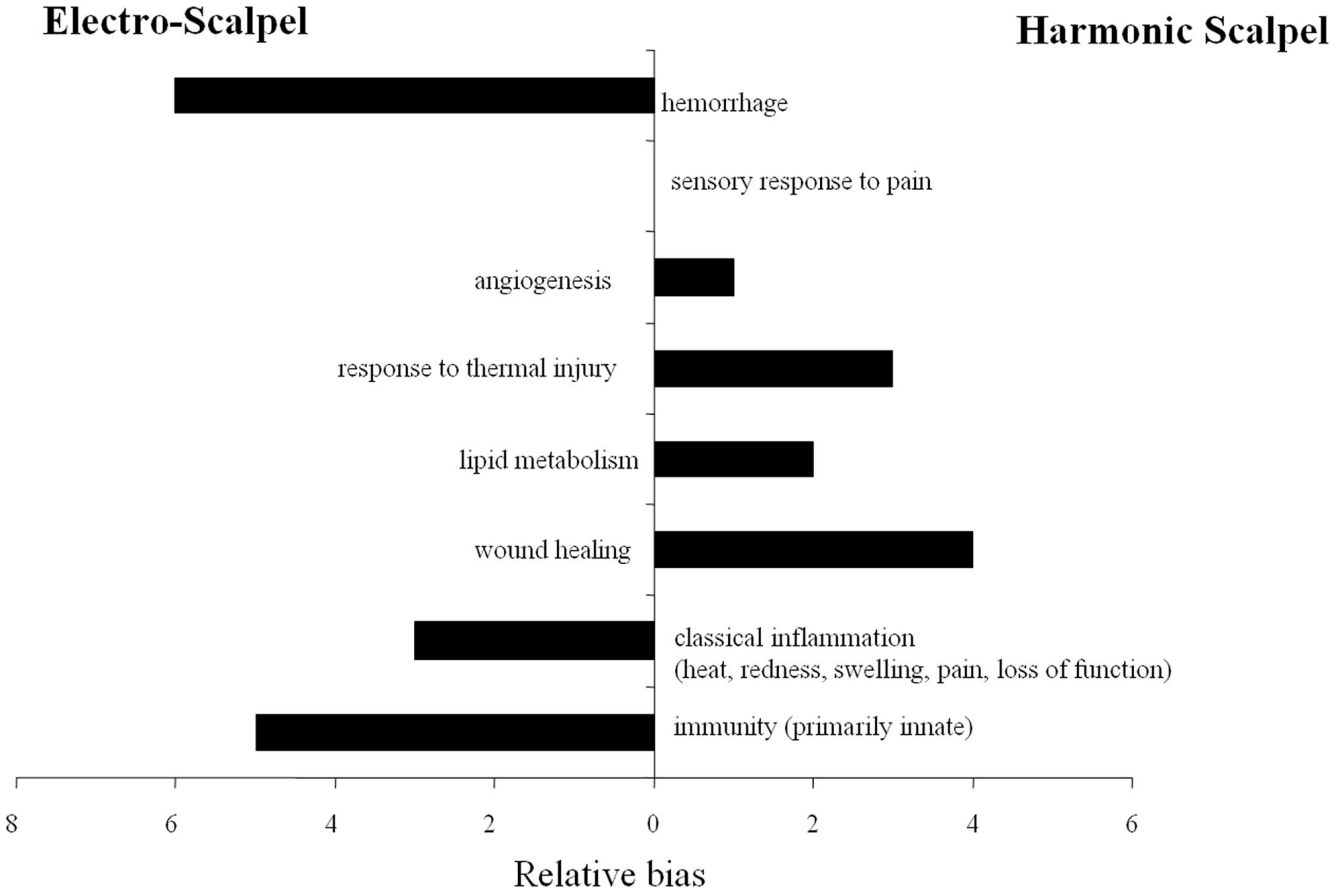
Comparison of the overall effect of the identified protein expression changes in response to harmonic blade and electrosurgery incisions on biological processes of relevance to wound healing using GOModeler workflow. We used Gene Ontology terms to represent specific biological processes, and identify the known effect on a function under consideration such as ‘positively regulates’ or ‘negatively regulates’.

A total of 968, 758 and 932 proteins were identified from subcutaneous fatty tissue control, ES-incised subcutaneous fatty tissue and HB-incised subcutaneous fatty tissue samples respectively by tandem mass spectrometry (Tables S2A–C). Relative to control 368 proteins were significantly differentially expressed in response to ES and 314 proteins with HB incision (tables S3A–B). As was done with DE genes, we conducted biological modeling of DE proteins using GO and IPA pathways analysis. Even at the protein level, GO based analysis identified changes in proteins involved in innate immunity, inflammation, wound healing, lipid metabolism angiogenesis and hemorrhage (Figure 5). At the protein level, ES and HB affected expression of similar number of proteins. The net bias on a given GO functional category as evaluated by GOModeler analysis revealed positive regulation of wound healing, response to thermal injury in HB-incised tissue (Figure 6). Proteins with significant increase in expression included several hemoglobin and immunoglobulin fragments (Tables 2, 3). There were significantly more differentially expressed proteins associated with hemorrhage, inflammation and immunity within tissue incised with ES. However, HB-incised tissue contained significantly more differentially expressed proteins associated with angiogenesis, response to thermal injury, lipid metabolism and wound healing.

**Table 2:** Proteins with significant changes in expression in response to electrosurgery and harmonic blade incisions.

Accession*	Gene Symbol	Protein Name	ES/C	HB/C
NP_999553	APOA4	apolipoprotein A-IV precursor	−4.0	4.4
1QPW_D	HBB	Chain D, Hemoglobin	−3.9	2.7
P02067	HBB	Hemoglobin subunit beta	−3.9	1.9
NP_001038055	PLG	plasminogen precursor	−3.8	2.7
NP_999612	HBE1	hemoglobin subunit epsilon	−3.8	2.5
NP_001123430	SERPINC1	antithrombin-III precursor	−3.7	3.2
1QPW_C	LOC100737768	Chain C, Hemoglobin	−3.6	2.5
NP_999174	C3	complement C3 precursor	−3.6	2.6
BAA07817	---	fibrinogen A-alpha-chain	−3.3	1.5
NP_999563	APOA1	apolipoprotein A-I preproprotein	−3.3	1.8
NP_001116549	ASPA	aspartoacylase	−3.1	−1.0
NP_001005208	ALB	serum albumin precursor	−2.7	−1.1
NP_001090918	C6	complement component C6 precursor	−2.5	−0.9
XP_001924401	IL17F	PREDICTED: interleukin-17F	−2.0	−1.6
NP_999118	HPX	hemopexin precursor	−1.8	−1.1
P02543	VIM	Vimentin	−1.7	−0.3
NP_001093400	FASN	fatty acid synthase	−1.4	−1.2
Q28833	VWF	von Willebrand factor	−1.3	−1.7
NP_001002817	FABP4	fatty acid-binding protein, adipocyte	−1.3	−1.7
NP_999535	ADIPOQ	adiponectin precursor	−0.9	−3.5
NP_001107174	SLA-2	MHC class I antigen 2 precursor	−0.5	−1.3
NP_001093401	FABP3	fatty acid-binding protein, heart	0.5	−1.3
CAP45900	CD79A	CD79-alpha protein	1.6	−3.1
NP_001093394	FGFR2	fibroblast growth factor receptor 2 precursor	1.6	−1.8
BAG12993	---	MHC class II antigen	4.6	−0.5
AAM76076	---	immunoglobulin kappa light chain VJ region	4.7	4.1
1006222A	---	hemoglobin beta 115–146	5.0	3.0
AAA52216	---	Ig gamma 1b chain constant region	5.3	−2.3
ABY64539	---	fibrinogen	5.5	4.7
NP_998993	LOC396781	IgG heavy chain precursor	5.9	1.6
AAA52218	IGG2B	Ig gamma 2b chain constant region, partial	6.2	1.9
ACD64981	---	immunoglobulin gamma-2 heavy chain constant region	6.6	5.4
ABY85808	---	immunoglobulin gamma chain 5b	7.7	−3.5

*Genbank or Uniprot accessions.

**Table 3:** Proteins with significant changes in expression in response to either electrosurgery or harmonic blade incisions.

Accession*	Gene Symbol	Protein Name	ES/C	HB/C
NP_001092055	AP3B1	AP-3 complex subunit beta-1	n.s.^#^	2.0
AAA74655	APOB	apolipoprotein B	n.s.	−3.8
NP_001002801	APOC3	apolipoprotein C-III precursor	n.d^$^	2.0
NP_999473	APOE	apolipoprotein E precursor	n.d	4.1
AAW02948	FN1	fibronectin, partial	n.s.	0.7
650438A	---	hemoglobin alpha	n.d	4.8
P01846	---	Ig lambda chain C region	n.s.	−3.1
ABY85806	---	immunoglobulin gamma chain 4b	n.s.	−1.5
ABY85809	---	immunoglobulin gamma chain 5a	n.s.	3.1
XP_001926353	MFI2	PREDICTED: LOW QUALITY PROTEIN: melanotransferrin	n.s.	1.8
AAA67022	---	Ig heavy chain variable VDJ region, partial	5.9	n.s.
AAA03520	---	Ig kappa chain, partial	9.3	n.s.
AAN07167	---	immunoglobulin delta heavy chain membrane bound form	4.7	n.s.
CAJ45403	---	immunoglobulin heavy chain variable region	4.7	n.s.
AAM76080	---	immunoglobulin kappa light chain VJ region	4.8	n.s.
NP_001116561	C4	complement C4 precursor	−3.4	n.s.
NP_001001646	C5	complement C5 precursor	−2.5	n.s.
NP_999332	F8	coagulation factor VIII precursor	−2.6	n.s.
AAR99598	FGG	fibrinogen gamma polypeptide, partial	−5.7	n.s.
NP_001093405	IKBKB	inhibitor of nuclear factor kappa-B kinase subunit beta	−3.2	n.s.
NP_999262	IL12RB2	interleukin-12 receptor subunit beta-2 precursor	−5.2	n.s.
NP_001230563	IL13RA2	interleukin-13 receptor subunit alpha-2 precursor	1.8	n.s.
NP_001070681	MIF	macrophage migration inhibitory factor	−2.2	n.s.
NP_001090913	TLR3	toll-like receptor 3 precursor	−4.3	n.s.
ACE62926	TLR4	toll-like receptor 4	−5.4	n.s.
NP_001106510	TLR4	toll-like receptor 4 precursor	−6.4	n.s.
NP_001116674	TLR5	toll-like receptor 5 precursor	−3.6	n.s.
NP_999171	CD9	CD9 antigen	−4.8	n.d
NP_999446	CFH	complement factor H precursor	−4.5	n.d
NP_001092053	KRT2	keratin, type II cytoskeletal 2 epidermal	1.7	n.d
NP_999527	LTF	lactotransferrin precursor	−3.4	n.d
AAT98289	---	T cell receptor beta	6.3	n.d
AAN06824	TXNRD1	thioredoxin reductase	−4.6	n.d
NP_001231465	THBS1	thrombospondin 1 precursor	−4.5	n.d

* Genbank or Uniprot accessions.

# n.s. indicates that there was no significant change in protein expression.

$ n.d. indicates that the protein was not identified in the dataset.

### Pathways analysis

Molecular functions and signaling pathways represented by the DE genes and proteins identified in porcine tissue in response to ES and HB incisions were identified by conducting Ingenuity pathways analysis. At the RNA level four of the top five molecular functions represented by DE genes were common to both datasets and were Cellular Growth and Proliferation, Cell Death, Cellular Movement, and Cellular Development. The top five signaling pathways identified in ES were PPARα/RXRα Activation, Aryl Hydrocarbon Receptor Signaling, TREM1 Signaling, Acute Phase Response Signaling, and Valine, Leucine and Isoleucine Degradation. In the HB dataset TREM1 Signaling, Coagulation System, Glucocorticoid Receptor Signaling, LXR/RXR Activation, and VDR/RXR Activation were identified as top five signaling pathways. The top five functions in HB were TREM1 Signaling and Glucocorticoid Receptor Signaling and in ES they were Hepatic Fibrosis/Hepatic Stellate Cell Activation and Acute Phase Response Signaling. At the protein level, of the top five functions identified in each data set four were common and included Cell Death, Cell-To-Cell Signaling and Interaction, Cellular Growth and Proliferation and Cellular Movement. Three of these functions excluding cell death were also observed at the RNA level. The top five signaling pathways identified had four in common to both, and included Acute Phase Response Signaling, Coagulation System, Fatty Acid Elongation in Mitochondria and Pyruvate Metabolism. While LXR/RXR Activation made up the top five signaling pathways in the ES dataset, Valine, Leucine and Isoleucine Degradation were identified amongst the top five signaling pathways in response to HB incision.

## Discussion

Wound healing follows a progression from hemostasis, to inflammation, then proliferation and finally remodeling. At the timepoint monitored in this study, three days after the incisional trauma was inflicted, the observed regulation of gene transcripts shows that the healing response was primarily in the inflammation phase during which an influx of neutrophils followed by macrophages occurs, whereas the proteins identified showed some evidence of inflammation but also the remnants of the hemostasis stage. For both these early phases of wound healing, ES incision produced a more severe response than HB.

A survey of the up-regulated transcripts shows a number of CXCL chemokines (CXCL2, CXCL4, CXCL6, CXCL7 and CXCL8) indicating a strong immune response (Table 1). Expression of these chemokines is relatively higher in response to ES incisions than in HB incisions. CXCL2 (MIP-2α) is secreted by macrophages and is chemotactic for polymorphonuclear leukocytes. CXCL4, also called platelet factor 4 (PF4), is released by platelets during blood coagulation, and is chemotactic for neutrophils, fibroblasts and monocytes. Similar regulation of CXCL2 and CXCL4 was observed in porcine skin following sulfur mustard and thermal burn injury [8]. CXCL6 (GCP-2) is chemotactic for neutrophilic granulocytes. Little has been reported on CXCL6 in response to thermal injury, but it is known to play a key role in surgical inflammation [12] and neutrophils are one of the early response cells in wound healing. Like CXCL4, CXCL7 or pro-platelet basic protein is released by platelets after activation, and is a chemoattractant for neutrophils. CXCL8, more commonly called interleukin-8 (IL-8), is produced by macrophages, endothelial cells and a variety of other cell types, and is a potent inflammatory agent and chemoattractant principally for neutrophils. While we are not aware of any studies on the effect of thermal injury on IL-8, even mild heating can induce its up-regulation [13]. Sterile trauma to the dermis has also been shown to increase levels of IL-8, which has been described as representing an innate response to “danger” [14].

Arginase-1, like the chemokines, had higher expression relative to control in the ES incisions than in HB incisions. Arginase is the final enzyme of the urea cycle, catalyzing the breakdown of arginine into ornithine and urea. Arginine is an essential substrate for the wound healing process and is intimately involved with cell signaling through the production of nitric oxide and cell proliferation through its metabolism to ornithine and the other polyamines [15]. Substantial regulation of arginase has been observed in porcine thermal injury [8] and chronic ischemic wounds [16].

Several other up-regulated genes are known to be part of the immune/inflammatory response, or play a critical role in the wound healing response. SerpinB2, usually described as a plasminogen activator inhibitor, also plays a key role in the adaptive immune response [17]. SerpinB2 has been found in keratinocytes during re-epithelialization of dermal burn wounds [18]. Tenascin is an extracellular matrix glycoprotein that can bind to fibronectin during wound healing. Enhanced expression of tenascin has been observed in keloid scar tissue recovering from burns at markedly higher levels than in healthy skin tissue [8],[19]. MARCO is a macrophage scavenger receptor and serves as part of the innate immune system, binding to both Gram-negative and Gram-positive bacteria. It may play a role in clearing apoptotic cells and debris from the injury site [20]. Interleukin-6 is both a pro-inflammatory cytokine secreted by T cells and macrophages, and is frequently used as a marker of the burn wound inflammatory response [8],[21].

The role that down-regulated genes (table S1C–D) play in wound response is not as clear. Members of the paraoxanase family prevent oxidative stress and fight inflammation [22], so it is reasonable that PON3 is decreased more by ES (−39.2 fold). Angiotensin is primarily known for its role in the control of systemic blood pressure, but recently additional functions have been found in wound healing [23]. Lower levels of angiotensin, as observed for ES (−21.6 fold), may be expected to delay the wound healing response. Carbonic anhydrases catalyze the conversion of CO_2_/H_2_0 to HCO_3_^−^/H^+^, helping to maintain acid-base balance. Decreased levels of carbonic anhydrase-3 gene transcripts were observed in this study with electrosurgery incisions, as has been seen for carbonic anhydrase protein isoforms in recalcitrant keloid scar tissue [24]. The marginal increase (1.29 fold, table S1C) in the expression of epidermal growth factor (EGF) at this early timepoint with elcterosurgery may not be sufficient to contribute to wound healing process. EGF has been shown to accelerate the rate of healing of partial-thickness skin wounds [25], and so would be expected to increase during healing.

These changes in individual transcript levels are reflected in the overall functional analysis. The transcript functional groups of immunity (primarily innate) and inflammation (including genes related to the classic signs of heat, redness, pain and loss of function) were associated more significantly with the ES incisions than those made with HB. This finding on the gene level is consistent with comparisons of ES and HB via macroscopic observations of immune response and inflammation [4],[5].

As with the gene transcripts, almost all of the most highly regulated proteins had higher expression in ES compared to HB incisions. Many of these proteins are remnants of the initial trauma and hemostatic process. Various fragments of hemoglobin alpha, beta and epsilon were observed along with fibrinogen and ABO system transferase. The response of the immune system is reflected in the appearance of several immunoglobulin components (IgM, IgA and IgG) and complement 3. On the other end of the spectrum, those proteins exhibiting decreased levels relative to control were present at lower levels in ES incisions than in HB. These decreased levels of proteins may be associated with impaired wound healing. Examples of diminished proteins include vimentin, an intermediate filament found in connective tissue that is critical to wound healing [26], and the iron-binding glycoprotein transferrin and albumin which are both associated with fewer wound healing complications [27].

Functional analysis of proteins showed some similarity to that of genes, namely immune response and inflammation were associated more with ES than with HB. In addition, hemorrhage was identified more closely with ES in agreement with the accepted macroscopic observation that HB provides better hemostatic control than ES [3],[28]. Functional analysis also showed higher significance in wound healing and angiogenesis for HB than ES, which has recently been corroborated in a preclinical model [5].

The results from the current study suggest that ES incisions produce more substantial effects at both the gene transcript level and the protein level than HB. At three days after surgery, tissue incised with HB exhibited lower levels of hemostasis remnants, fewer inflammation and immune-related genes and proteins, and more advanced wound healing/angiogenesis than ES. These differences are consistent with observations made in pre-clinical models [4]–[6]. In clinical studies, reduced pain, perhaps as a consequence of reduced inflammation, has been observed in a variety of surgical procedures [29]–[31].

The reasons for the differences between the devices observed in this study were not determined. In addition to having a different mechanism of action (mechanical action vs the passage of current), HB also produces less heat and thermal injury than ES [32]. Conclusions drawn from this study are limited by several factors including the species (pig) and tissue type (subcutaneous fat), the timing of the samples (single timepoint at 3 days post-surgery) and sensitivity of the protein assay. Further work would benefit from monitoring gene and protein levels at both earlier and later timepoints in the wound healing process, and examining other tissue types, such as muscle or connective tissue.

## Conclusions

In this study we showed that changes in gene transcript and protein levels after incisions with energized devices were observable at three days after surgery, and that differences exist between energized devices. The HARMONIC® Blade appeared to produce better hemostasis, less inflammatory/immune response, and more advanced wound healing than electrosurgery. These differences in the preclinical model may be responsible for the advantages observable in clinical studies, such as less blood loss and reduced pain with HARMONIC® Blade in comparison to electrosurgery.

## Methods

### Animals

The study was reviewed and approved by the Institutional Animal Care and Use Committee of Ethicon Endo-surgery, Inc.

Two 4-month old female pigs weighing 40–55 kg were used in this study. Pigs were pre-anesthetized with 0.5 mg/kg acepromazine and 0.01 mg/kg glycopyrrolate administered intramuscularly (i.m.). Anesthesia was induced with 2 mg/kg xylazine i.m. and maintained with isoflurane at 1.5–2.0%. Pre-operatively, the animal received 22 mg/kg Cephazolin (WG Critical Care Parmus, NJ) IV during surgical prep and buprenorphine at 0.01 mg/kg subcutaneously. A 100 µg/hr fentanyl patch (Butler ScheinAnimal Health, Dublin, OH) was placed prior to the beginning of surgery.

### Surgical Treatment & Tissue Processing

Each animal had four 4-cm midline caudal abdominal incisions created with the test devices. The test devices were monopolar electrosurgery (Electrocautery Pencil, ConMed, Utica NY) and HARMONIC® Blade (HK105, Ethicon Endo-Surgery, Cincinnati OH). Each animal had two HB incisions and two ES incisions for a total of 8 incisions.

Each incision was started with a scalpel blade through the skin (epidermis and dermis), and then deepened through the subcutaneous tissues down to the linea alba using either HB at power level 5 for cutting tissue and power level 3 for coagulating bleeders, or ES set at 40 Watts for cutting and Blend 2/Spray for coagulation. The linea alba was not incised. Closure of the incision was accomplished by apposing the skin with 3-0 nylon in a simple interrupted pattern. DERMABOND® Topical Skin Adhesive was then applied to seal the epidermis. No bandages were applied to the incisions. All animals recovered uneventfully.

After three days, incision sites were surgically removed using a cold steel scalpel en bloc from the abdominal wall from anesthetized animals. The tissue block was sliced perpendicular to the surgically incision to create slabs 4–6 mm thick. The slab was trimmed to remove the skin from the top and muscle from the bottom of the sample. The side walls were trimmed to approximately 4 mm from the surgical incision leaving a rectangular block of subcutaneous fatty tissue. Four to six samples were taken from each incision. Animals were then euthanized.

Samples for genomics and proteomics analysis were placed in sterile DNAse-, RNAse-, protein-free Eppendorf tubes, flash frozen in liquid nitrogen, held on dry ice throughout tissue collection, and then stored at −80°C.

### Histopathology analysis

Samples for histological analysis, corresponding to the same locations and times as for the genomics and proteomics analysis, were collected into 10% neutral buffered formalin. The samples were trimmed, processed routinely, embedded in paraffin, sectioned at approximately 5 microns, and stained with hematoxylin and eosin (H&E) stain. Slides were evaluated by a veterinary pathologist. Where appropriate, microscopic findings were given a severity score, where 1 = minimal, 2 = mild, 3 = moderate, and 4 = severe.

### RNA isolation, microarrays and data analysis

Total RNA was isolated in quadruplicate from 100 mg of tissue (four each of control, ES, HB). Briefly, frozen tissue samples were pulverized in liquid nitrogen and Qiazol® buffer (Qiagen, Germantown MD; 500 µL per sample) and then further homogenized using Qiashredder columns (Qiagen). RNA was purified using the RNase-Free DNase Set and the RNeasy Lipid Tissue Mini Kit (Qiagen). Isolated RNA was quantified spectrophotometrically (NanoDrop™ 1000; Thermo Scientific, Waltham MA) and RNA quality measured (2100 Bioanalyzer and RNA 6000 Nano chips, Agilent Technologies, Santa Clara CA). Only RNA with high integrity (RIN>8.0; 28S/18S>2.0) and concentration (>800 ng/µL) was used in microarray experiments and RNA samples were repurified until each exceeded these standards. Purified RNA was labeled using the One-Cycle Eukaryotic Target Labeling Assay (Affymetrix, Santa Clara CA) [33]. Labeled RNA was hybridized to the GeneChip® Porcine Genome Arrays (Affymetrix) which contain 23,937 probe sets to interrogate 23,256 transcripts in pig. The arrays were scanned at the Fluidics Station 400 (Affymetrix) and microarray expression data were generated with GeneChip® Operating Software (GCOS; Affymetrix) [34]. Log transformed raw intensity values for all chips were normalized using RMA [35] in Affymetrix expression console. Differential mRNA expression analysis used t-tests within the Spotfire® software (Tibco Software, Somerville, MA) and multiple testing corrections using the Benjamini-Hochberg method [36]. The microarray data compliant with MIAME guidelines was submitted to The Gene Expression Omnibus (GEO) database [37] at the National Center for Biotechnology Information (NCBI). (Accession numbers pending).

Although the microarray used is a commercial product, most of the probes are not identified as a specific gene; this is obviously a significant impediment to identifying the pathophysiology in the tissues later. It was first necessary to identify which genes are represented by each differentially-expressed probe. We did so by both mapping probes to pig and also human accession numbers in the nucleotide and expressed sequence tag databases as well as by reciprocal BLASTx searches to Refseq pig proteins, and proteins from the genome sequencing project for Sus scrofa [38]. All of these accessions were updated (May 1,2013) in this submission.

### Proteomics

Total protein was isolated in quadruplicate from tissue (four each of control, ES, HB). The samples were weighed and weights were normalized relative to that of the smallest sample. Proteins were isolated using differential detergent fractionation (DDF) [39]. Total proteins (from 350–450 mg tissue) were reduced with 5 mM DTT at 65°C for 5 min and alkylated with 10 mM iodoacetamide at 30°C for 30 min. Trypsin digestion used molecular biology grade porcine trypsin (5 µg; 37°C; 16 h; 50∶1 ratio of protein:trypsin; Promega Corporation, Madison, WI). After digestion all samples were adjusted to 2% acetonitrile (ACN) and desalted using a peptide macrotrap (Michrom BioResources Inc., Auburn, CA TR1/25108/52). Following desalting, all samples were dried and resuspended in 20 microliters of 5% acetonitrile, 0.1% formic acid (FA) and transferred to low retention HPLC vials.

HPLC columns used in this experiment for peptide separation were BioBasic C18 reversed phase columns (Thermo 72105-100266). The mass spectrometer (Thermo Finnigan LCQ DECA XP Plus with a Thermo nanospray type 1 ion source) was equipped with a 10-port valve which can accommodate two columns so that one column can be loaded as the other is eluted to minimize run time. Prior to each HLPC sample run, both columns are washed with 100% methanol for 30 minutes and then equilibrated with 5% acetonitrile ACN, 0.1% FA for 90 minutes. During the equilibration process, the nanospray needle (New Objective FS360-75-8-N-5-C12) and ion transfer capillary were cleaned with methanol and water for 30 minutes. Afterward the mass spectrometer was tuned using the Angiotensin (in 27% ACN, 0.1% FA) m/z of 433.3 for 60 minutes at which time the equilibrated columns and the HPLC system (Thermo Surveyor system) were ready for operation.

One machine control (not the study control) and sample were run 12-hourly as part of a programmed sequence. The machine control digest (bovine serum albumin, equine cytochrome C and myoglobin, and yeast enolase, 2 picomoles each) was manually-loaded using a pressure bomb onto one reverse phase column and equilibrated for 20 minutes using 5% ACN, 0.1% FA; all HPLC solutions used contain 0.1% FA. Once the control was loaded, the 10-port valve was rotated and the control was eluted for online ms/ms analysis while the sample peptides were loaded on the second RP column. The elution gradient for the control consists of 5% ACN to 50% ACN in 65 minutes followed by a 20 minute wash with 95% ACN and a 25 minute equilibration using 5% ACN. The flow rate was 500 nanoliters per minute for all HPLC runs. Peptide separation for each sample was achieved using a 5% to 25% ACN gradient in 450 minutes followed by 25% to 50% ACN gradient in 130 minutes. The gradient was followed by a 20 minute wash with 95% ACN and equilibration with 5% ACN for 25 minutes. Data was collected for 625 minutes over the duration of each HPLC run using repetitive MS scans immediately followed by three MS/MS scans of the three most intense MS peaks. Dynamic exclusion was enabled with duration of two minutes and a repeat count of two. The same scan parameters were also used for the control. Ionization of peptides was achieved via nanospray ionization using a Thermo Finnigan nanospray source type I operated at 1.9 kV with 8 micrometer internal diameter silica tips (New Objective FS360-75-8-N-20-C12). High voltage was applied using a t-connector with a gold electrode in contact with the HPLC solvent.

### Protein identification

All searches were done using TurboSEQUEST™ (Bioworks Browser 3.3; ThermoElectron, Cluster version). Mass spectra and tandem mass spectra were searched against an *in silico* trypsin-digested pig protein database. We created a FASTA file for searching the proteome that had Refseq pig proteins and proteins from the pig genome project. Cysteine carboxyamidomethylation and methionine single and double oxidation were included in the search criteria. We used reverse database strategy (using the reverse function in Bioworks) to estimate the probability for peptide identification using custom Perl scripts and a Sequest-based method [40]. The reversed database was *in silico* trypsin digested and used for searches with tandem mass spectra as described above. Proteins identified with peptides identified at p≤0.05 and at least 6 amino acids length were accepted.

We used the computationally-intensive “Monte Carlo” statistical method or random resampling with replacement to analyze quantitative changes in proteins based on ∑Xcorr [41]. We used *Protquant*, a variant of spectral counting for relative quantification [9] to impute the missing Xcorr values and used custom perl scripts tool for resampling with replacement and p value calculation. We performed 10,000 replications for stable estimation of p values. To correct for multiple testing, we determined the false discovery rate (FDR) for p value using the Benjamini-Hochberg method [36]. Fold changes in protein expression were calculated as described earlier [42]. The proteomics data was submitted to PRIDE database [43], which is a public repository that is compliant with proteomics community standards for proteomics data, including protein and peptide identifications. The ProteomeXchange accession number assigned to our datasets is PXD000038. Accession numbers (GI) for all identified proteins were updated to reflect the latest version at Genbank/Uniprot databases (April 26, 2013).

### Functional Modeling of significant changes in gene and protein expression

All mRNAs and proteins that we identified as differentially-expressed between the ES and HB samples were then identified by statistical comparison of the differences between each of the treatments and the control (again by Monte-Carlo resampling followed by Benjamini-Hochberg correction). The workflow for network/pathway modeling and Gene Ontology based hypothesis testing was identical for mRNA and protein and is described below.

To gain insights into the biological pathways and networks that are significantly represented we used Ingenuity Pathways Analysis (IPA®, Ingenuity Systems, Redwood City CA). Currently IPA does not accept gene/protein accession numbers from pig. Therefore we mapped porcine identifiers from our datasets to their corresponding human orthologs using Inparanoid and GOAnna [44]. We built networks from the list of differentially-expressed genes and proteins that had human orthologs in IPA and selected networks scoring ≥2, which have >99% confidence of not being generated by chance for further analysis as described earlier [45]. Biological functions for genes in each network are based on annotations from scientific literature that are stored in the IPA knowledge base. The Fisher exact test is used to calculate the p-value determining the probability of each biological function/disease or pathway being assigned by chance. We used p≤0.05 to select highly significant biological functions and pathways represented in our microarray datasets [39].

GO-based modeling was based on the specific hypotheses, framed in synonymous GO biological process terms, defining the pathophysiological phenotypes relevant to the wound healing process. To derive quantitative data we combined GO-annotation with our quantitative mRNA or protein expression data. We first identified all of the differentially-expressed gene products (i.e. mRNAs and proteins) that already had functional-annotations available in our AgBase® databases. For gene products that did not have functional-annotations, we functionally-annotated these from published literature using the processes described [46]. We next used our GO modeler algorithm [47], which first scores the effects of each gene product biological process annotation as either pro, anti, no effect or no data. Then the quantitative mRNA or proteomics data, as appropriate, is used to calculate a quantitative effect for each gene (i.e. to give a quantitative value in each cell). Finally, net effects were calculated for both the mRNA and the protein data.

## Supporting Information

Table S1 **Porcine gene response following incision with energized devices.**
(XLS)

Table S2 **Total number of proteins and the corresponding number of peptides identified in control dataset.**
(XLSX)

Table S3 **Supplementary** **table 3A** **. Proteins identified as differentially expressed in response to electrosurgery incision.**
(XLSX)
